# c-Myb knockdown increases the neomycin-induced damage to hair-cell-like HEI-OC1 cells in vitro

**DOI:** 10.1038/srep41094

**Published:** 2017-01-23

**Authors:** Xiaoyu Yu, Wenwen Liu, Zhaomin Fan, Fuping Qian, Daogong Zhang, Yuechen Han, Lei Xu, Gaoying Sun, Jieyu Qi, Shasha Zhang, Mingliang Tang, Jianfeng Li, Renjie Chai, Haibo Wang

**Affiliations:** 1 Otolaryngology-Head and Neck Surgery, Shandong Provincial Hospital Affiliated to Shandong University, Jinan, China; 2 Shandong Provincial Key Laboratory of Otology, Jinan, China; 3 Key Laboratory of Developmental Genes and Human Disease, Ministry of Education, Institute of Life Sciences, Southeast University, Nanjing 210096, China; 4 Co-innovation Center of Neuroregeneration, Nantong University, Nantong 226001, China; 5 Department of Otolaryngology Head and Neck Surgery, Zhongda Hospital, Southeast University, Nanjing 210096, China

## Abstract

c-Myb is a transcription factor that plays a key role in cell proliferation, differentiation, and apoptosis. It has been reported that c-Myb is expressed within the chicken otic placode, but whether c-Myb exists in the mammalian cochlea, and how it exerts its effects, has not been explored yet. Here, we investigated the expression of c-Myb in the postnatal mouse cochlea and HEI-OC1 cells and found that c-Myb was expressed in the hair cells (HCs) of mouse cochlea as well as in cultured HEI-OC1 cells. Next, we demonstrated that c-Myb expression was decreased in response to neomycin treatment in both cochlear HCs and HEI-OC1 cells, suggesting an otoprotective role for c-Myb. We then knocked down c-Myb expression with shRNA transfection in HEI-OC1 cells and found that c-Myb knockdown decreased cell viability, increased expression of pro-apoptotic factors, and enhanced cell apoptosis after neomycin insult. Mechanistic studies revealed that c-Myb knockdown increased cellular levels of reactive oxygen species and decreased Bcl-2 expression, both of which are likely to be responsible for the increased sensitivity of c-Myb knockdown cells to neomycin. This study provides evidence that c-Myb might serve as a new target for the prevention of aminoglycoside-induced HC loss.

Hearing loss is one of the most common sensory disorders in humans, and it not only affects the life quality of the afflicted individuals, but also imposes a heavy social and economic burden on families and the community. While the exact prevalence is uncertain, it is estimated by the WHO that over 360 million people suffer from hearing loss globally^1^. In most situations, hearing loss is primarily caused by the degeneration of or damage to the mechanosensory hair cells (HCs) because HCs play an essential role in converting mechanical sound waves into neural signals for hearing. Therefore, the survival of HCs is indispensable for the preservation of hearing, and damage to HCs leads to irreversible hearing loss in mammals because the mammalian cochlear HCs are terminally differentiated and are unable to regenerate once damaged^2^,^3^,^4^. It is well known that these HCs are susceptible to death from aminoglycosides, non-steroidal anti-inflammatory drugs, noise, and aging. Aminoglycosides, such as gentamicin, amikacin, kanamycin, and neomycin, are widely used antibiotics in the treatment of gram-negative bacterial infections because they are cost effective. However, the ototoxicity of aminoglycosides is a significant obstacle to their wider clinical application. The generation of reactive oxygen species (ROS) is presumed to be a principal mechanism underlying the ototoxicity of aminoglycosides^5^,^6^. Excess ROS overwhelms the redox balance and skews cell metabolism toward the activation of intrinsic apoptosis, which is regulated by the combined actions of pro- and anti-apoptotic members of the Bcl-2 family^7^,^8^,^9^. It has been well established that the anti-apoptotic protein Bcl-2 can prevent the release of cytochrome c and reduce the activation of caspase-9 and caspase-3, thus inhibiting caspase-3–dependent apoptosis^10^.

c-Myb, a member of the Myb family (A-Myb, B-Myb, and c-Myb), is a highly conserved transcription factor that plays a key role in cell proliferation, differentiation, and apoptosis^11^,^12^. The importance of c-Myb in the regulation of cell fate has been confirmed in hematopoietic cells; epithelial cells of the colon, kidney, and mammary gland^13^; smooth muscle cells^14^; and neural stem cells^15^. In the hematopoietic system, c-Myb is down-regulated after cell differentiation^16^,^17^,^18^, while in the aged hippocampus of gerbils, c-Myb expression is significantly increased^19^. The suppression of c-Myb leads to cell cycle arrest at G1 phase and an increase in the proportion of cells undergoing apoptosis^20^. In contrast, the overexpression of c-Myb promotes cell cycle progression and prevents apoptosis^21^. The mechanism behind this activity involves positive regulation of the anti-apoptotic gene Bcl-2^13^,^22^,^23^,^24^ and negative regulation of ROS generation in cardiomyocytes^25^. In some previous experiments, the anti-apoptotic role of c-Myb was not immediately apparent because shRNA-mediated knockdown did not induce significant apoptosis by itself 26, but it did enhance cell sensitivity to chemical agents^13^.

It has been reported that c-Myb is present within the chicken otic placode – a specialized region that gives rise to the components of the inner ear – and that c-Myb plays a role in controlling the onset of Sox10 expression, which serves as a key marker for otic development^27^. However, the expression and function of c-Myb in the mammalian inner ear have not been explored. The present study was designed to determine the expression pattern of c-Myb in the mammalian inner ear and to investigate the function of c-Myb in HEI-OC1 cells.

## Results

### c-Myb is expressed in the cochlea in an age-dependent and cell-specific manner

**Figure 1:**
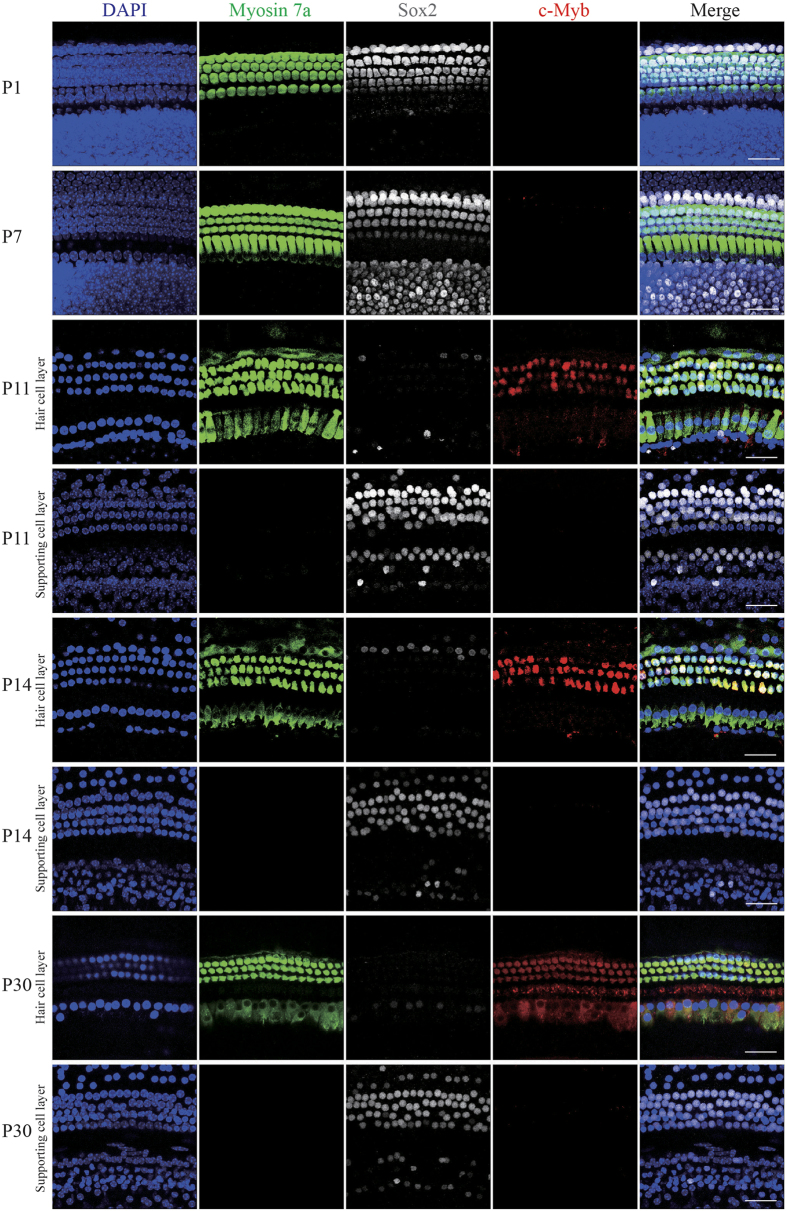
c-Myb expression in the cochlear hair cells. Immunofluorescence staining showed the expression pattern of c-Myb (red) in the postnatal cochlea. Myosin 7a (green) and Sox2 (grey) were used as HC and SC markers, respectively. Scale bars = 30 μm.

**Figure 2:**
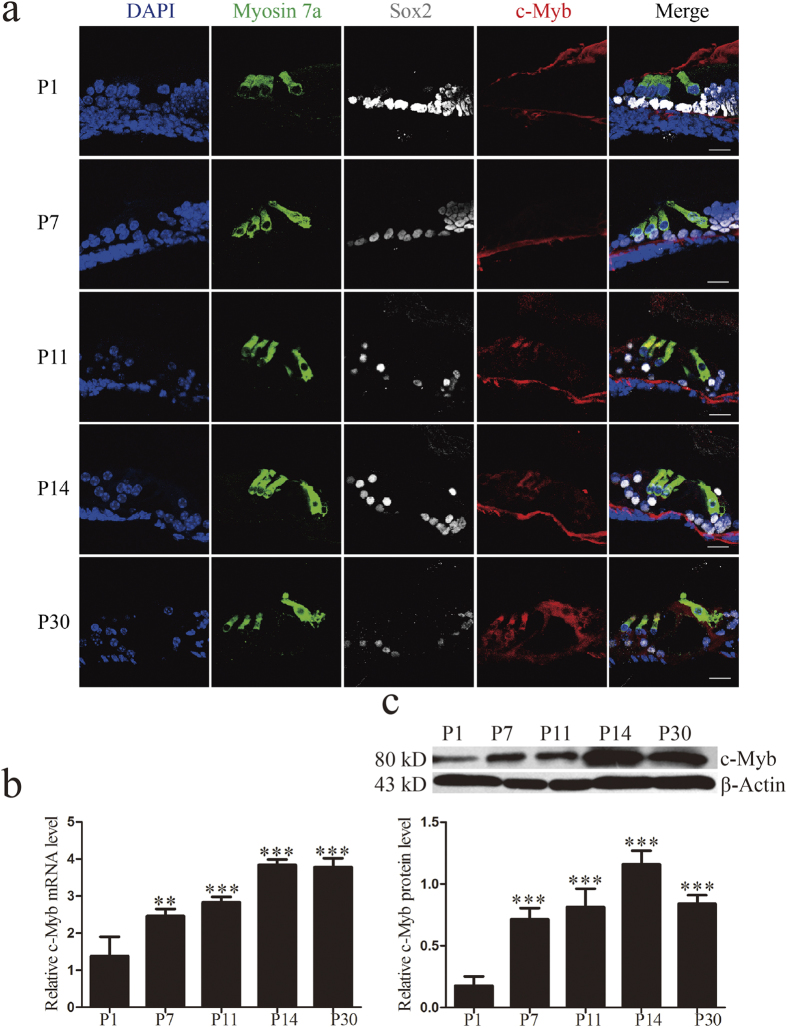
The expression level of c-Myb was up-regulated during later postnatal development. (**a**) Immunofluorescence staining in cryosections of the cochlea confirmed the expression pattern of c-Myb. (**b**) qRT-PCR results showed the mRNA levels of c-Myb at different time points. GAPDH was used as a housekeeping gene for control purposes. (**c**) Western blot analysis showed the protein levels of c-Myb at different time points. β-actin served as a loading control in each lane. **p < 0.01, ***p < 0.001, n = 3. Scale bars = 30 μm.

To characterize the expression pattern of c-Myb in the postnatal cochlea, we analyzed the inner ears of C57BL/6 mice at different ages. From postnatal day (P) 1 to P30, the pattern of c-Myb expression in the cochlear sensory epithelium changed (Figs 1 and 2). Because c-Myb had the same expression pattern in all three turns of the cochlea, we selected the middle turn as the representative sample. Myosin 7a and Sox2 were used as HC and supporting cell (SC) markers, respectively. Immunostaining showed that there was no expression of c-Myb in the P1 cochlea, and the expression of c-Myb was almost undetectable at P7 (Fig. 1). c-Myb expression was present at a low level specifically in outer HCs (OHCs) from P11, but not in inner HCs (IHCs) or SCs (Fig. 1). At P14, robust c-Myb expression was observed in the OHCs and faint expression was noted in the IHCs, but no expression was found in SCs (Fig. 1). From P30, c-Myb was strongly expressed in both OHCs and IHCs, but there was still no expression in SCs (Fig. 1).

Immunofluorescence staining was also performed on frozen cochlear cryosections to examine the expression pattern of c-Myb, and the same expression pattern was seen as in Fig. 1 (Fig. 2a). qRT-PCR and western blot were used to confirm the mRNA and protein expression levels, respectively, of c-Myb in the mouse cochlea. The results showed that both mRNA and protein levels of c-Myb were higher at P11 and that they reached their highest levels at P14 and P30 (**p < 0.01, ***p < 0.001, n = 3) (Fig. 2b,c). Taken together, these results demonstrated that c-Myb is expressed in an age-dependent and cell-specific manner in the postnatal organ of Corti.

### c-Myb is expressed in the HEI-OC1 cell line

**Figure 3:**
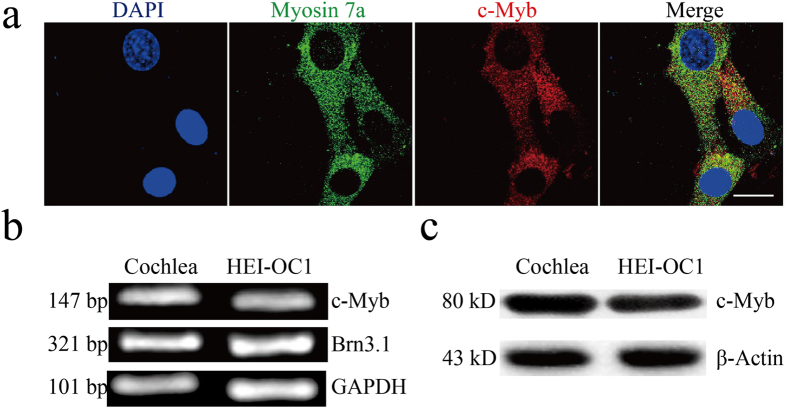
c-Myb expression in HEI-OC1 cells. (**a**) Immunofluorescence staining showed the expression of c-Myb (red) in HEI-OC1 cells. Myosin 7a (green) was used as the marker of HEI-OC1 cells. Scale bar = 5 μm. **(b**) RT-PCR results showed that c-Myb is expressed in the P14 mouse cochlea and in HEI-OC1 cells. Brn3.1 and GAPDH served as loading controls in each lane. (**c)** Western blot results confirmed that c-Myb is expressed in the P14 mouse cochlea and HEI-OC1 cells. β-actin served as the loading control in each lane.

HEI-OC1 cells, a HC-like cell line, express several molecular markers of cochlear HCs, including calbindin, calmodulin, math1, myosin7a, and prestin^28^,^29^,^30^,^31^, and they are commonly used to study the protection of HCs. We used Myosin 7a as the HC marker, and immunostaining showed that all HEI-OC1 cells could be double labeled by Myosin 7a and c-Myb, demonstrating that c-Myb is expressed in HEI-OC1 cells (Fig. 3a). RT-PCR and western blot analysis validated the mRNA and protein expressions of c-Myb in HEI-OC1 cells and in P14 cochleae (Fig. 3b,c).

### c-Myb expression in cochlear HCs is decreased after neomycin injury

**Figure 4:**
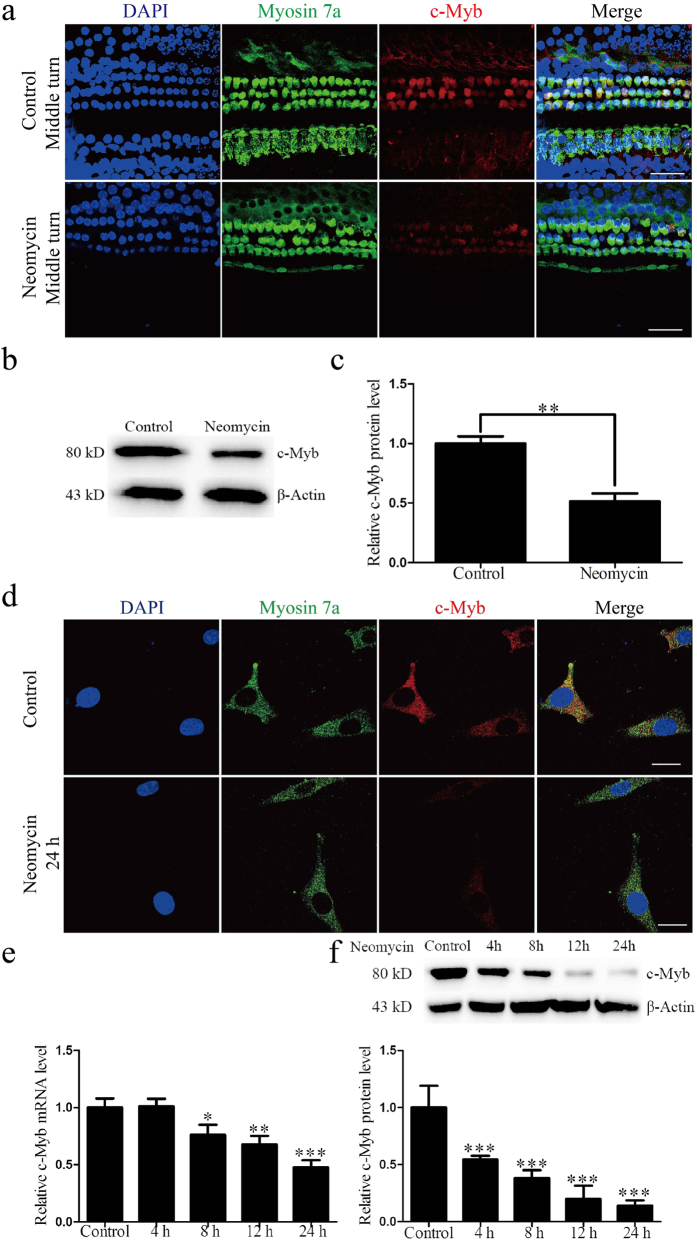
c-Myb expression was decreased in cochlear HCs and HEI-OC1 cells after neomycin exposure. (**a**) Immunofluorescence staining showed that c-Myb expression in cochlear HCs was decreased during neomycin treatment. Mice were given daily subcutaneous injections of neomycin (200 mg/kg) from P7 to P14. At P17, cochlear sensory epithelium samples were dissected out. Mice in the control group were given sterile saline. Scale bars = 30 μm. (**b** and **c**) The western blot analysis confirmed that the protein level of c-Myb in cochlear HCs was decreased during neomycin treatment. (**d**) Immunofluorescence staining in HEI-OC1 cells showed that the expression of c-Myb was decreased after neomycin treatment. The control group was HEI-OC1 cells without any treatment. Scale bars = 5 μm. (**e**) The mRNA level of c-Myb in HEI-OC1 cells was reduced with the increasing neomycin exposure time. GAPDH was used as a housekeeping gene for control purposes. (**f**) The protein level of c-Myb in HEI-OC1 cells decreased in a time-dependent manner. β-actin served as the loading control in each lane. *p < 0.05, **p < 0.01, ***p < 0.001, n = 3.

To determine whether the c-Myb expression in cochlear HCs was affected by neomycin treatment, C57BL/6 mice were given daily subcutaneous injections of neomycin (200 mg/kg) from P7 to P14, which is the ototoxic-sensitive period in the cochlea^32^. Mice in the control group were given sterile saline. At P17, the cochlear sensory epithelium was dissected out, and as reported before, the HC loss after neomycin injection forms a gradient from the apex to the base with the basal turns having greater HC loss than the apical turns^32^. We chose the middle turn as the representative image (Fig. 4a), and to better show the HC damage and the change of c-Myb expression over the whole cochlea we showed representative images of the basal and apical turns in Supplemental Fig. 1. Immunostaining showed that c-Myb expression was also decreased in the surviving HCs after neomycin treatment compared to the undamaged controls (Fig. 4a, Supplemental Fig. 1).

We performed a western blot to determine the protein level of c-Myb in cochlear HCs after neomycin insult, confirming that the protein level of c-Myb in cochlear HCs decreased after neomycin treatment (**p < 0.01, n = 3) (Fig. 4b,c). Taken together, these results demonstrated that neomycin injury led to a decrease in the expression of c-Myb in HCs, indicating that c-Myb might play an otoprotective role in normal cochlear HCs.

### The expression of c-Myb in HEI-OC1 cells is down-regulated after neomycin injury

To determine whether the expression of c-Myb in HEI-OC1 cells could be affected by neomycin damage, we treated the HEI-OC1 cells with 2 mM neomycin. Immunofluorescence staining with anti-c-Myb and anti-Myosin 7a antibodies showed that after 24 h neomycin treatment, the c-Myb expression was significantly reduced (Fig. 4d). qRT-PCR demonstrated that the mRNA level of c-Myb in HEI-OC1 cells was significantly decreased at 8 h after neomycin treatment and was further reduced as time went on (*p < 0.05, **p < 0.01, ***p < 0.001, n = 3) (Fig. 4e). The protein expression of c-Myb began to decline at 4 h after exposure to neomycin and continued to decrease to its lowest level at 24 h after exposure (***p < 0.001, n = 3) (Fig. 4f). These results showed that c-Myb expression in HEI-OC1 cells is down-regulated in a time-dependent manner after neomycin injury.

### Knockdown of c-Myb expression decreases HEI-OC1 cell viability after neomycin injury

**Figure 5:**
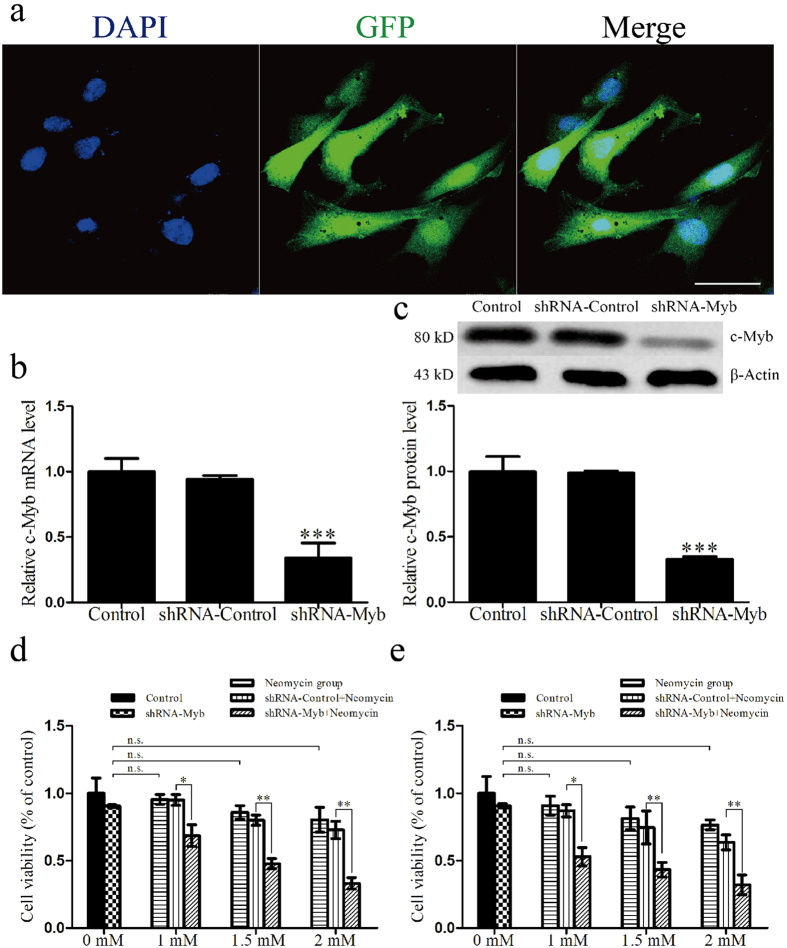
c-Myb knockdown decreased the HEI-OC1 cell viability after neomycin exposure. All control groups were HEI-OC1 cells without any treatment. (**a**) Representative images of GFP expression in cells transfected with nonsense shRNA conjugated with GFP at 48 h after transfection. (**b**) The mRNA levels of c-Myb in control, shRNA-Control, and shRNA-Myb groups. GAPDH was used as a housekeeping gene for control purposes. (**c**) The protein levels of c-Myb in control, shRNA-Control, and shRNA-Myb groups. β-actin served as the loading control in each lane. (**d**) At 48 h after shRNA transfection, the HEI-OC1 cells were treated with neomycin at final concentrations of 1 mM, 1.5 mM, and 2 mM for 24 h. We had five groups: the control groups had no treatment at all, the shRNA-Myb groups only received shRNA-Myb transfection but no neomycin, the neomycin group only received neomycin treatment, the shRNA-Control-neomycin group received nonsense shRNA transfection and neomycin treatment, and the shRNA-Myb-neomycin group received shRNA-Myb transfection and neomycin treatment. The MTT assay was carried out to detect cell viability. (**e**) At 48 h after shRNA transfection, the HEI-OC1 cells were treated with neomycin at final concentrations of 1 mM, 1.5 mM, and 2 mM for 48 h. The five groups were the same as before. The MTT assay was carried out to detect cell viability. For all experiments, the values for normal controls were set to 1. *p < 0.05, **p < 0.01, ***p < 0.001, n = 3. Scale bar = 10 μm.

In order to investigate the role of c-Myb in neomycin-induced cell death of HEI-OC1 cells, we knocked down c-Myb by shRNA transfection. In all experiments, the control group was HEI-OC1 cells without any treatment, and the shRNA-Control group was HEI-OC1 cells transfected with nonsense shRNA (shRNA-Control). First, we measured the efficiency of the transfection system using nonsense shRNA conjugated with GFP, and immunocytochemistry showed a successful transfection (Fig. 5a). qRT-PCR and western blot showed that the mRNA and protein levels of c-Myb were significantly decreased in the shRNA-Myb–transfected group (a decrease to 33.88 ± 11.53% and 32.67 ± 2.08% of the control, respectively) but remained unchanged in the shRNA-Control–transfected group (Fig. 5b,c). These results demonstrated that shRNA-Control transfection has no effect on c-Myb expression, while shRNA-Myb transfection effectively suppresses c-Myb expression in HEI-OC1 cells (***p < 0.001, n = 3).

At 48 h after shRNA transfection, the HEI-OC1 cells were treated with neomycin at final concentrations of 1 mM, 1.5 mM, and 2 mM for 24 h or 48 h. We had five groups: the control groups had no treatment at all, the shRNA-Myb groups only received shRNA-Myb transfection but no neomycin, the neomycin group only received neomycin treatment, the shRNA-Control-neomycin group received nonsense shRNA transfection and neomycin treatment, and the shRNA-Myb-neomycin group received shRNA-Myb transfection and neomycin treatment. The MTT assay was carried out to detect cell viability. We found that after both 24 h and 48 h culture without neomycin treatment, c-Myb knockdown slightly reduced the HEI-OC1 cell viability, suggesting that c-Myb inhibition itself can lead to the death of HEI-OC1 cells, but this reduction was not statistically significant. With neomycin treatment, cell viabilities in the shRNA-Myb group were significantly reduced compared to those of the shRNA-Control group, and higher concentrations of neomycin led to greater reductions in cell viability in HEI-OC1 cells with c-Myb inhibition (the cell viability in the shRNA-Myb group was reduced to 72.02 ± 6.51% and 45.95 ± 9.29% of the shRNA-Control group with 1 mM and 2 mM neomycin for 24 h, and 61.13 ± 8.87% and 50.00 ± 7.51% of the shRNA-Control group with 1 mM and 2 mM neomycin for 48 h; *p < 0.05, **p < 0.01, n = 3) (Fig. 5d,e), suggesting that neomycin injury enhances the c-Myb inhibition-induced cell death and that c-Myb inhibition makes the HEI-OC1 cells more sensitive to neomycin-induced damage.

### Knockdown of c-Myb expression increases HEI-OC1 cell apoptosis after neomycin exposure

**Figure 6:**
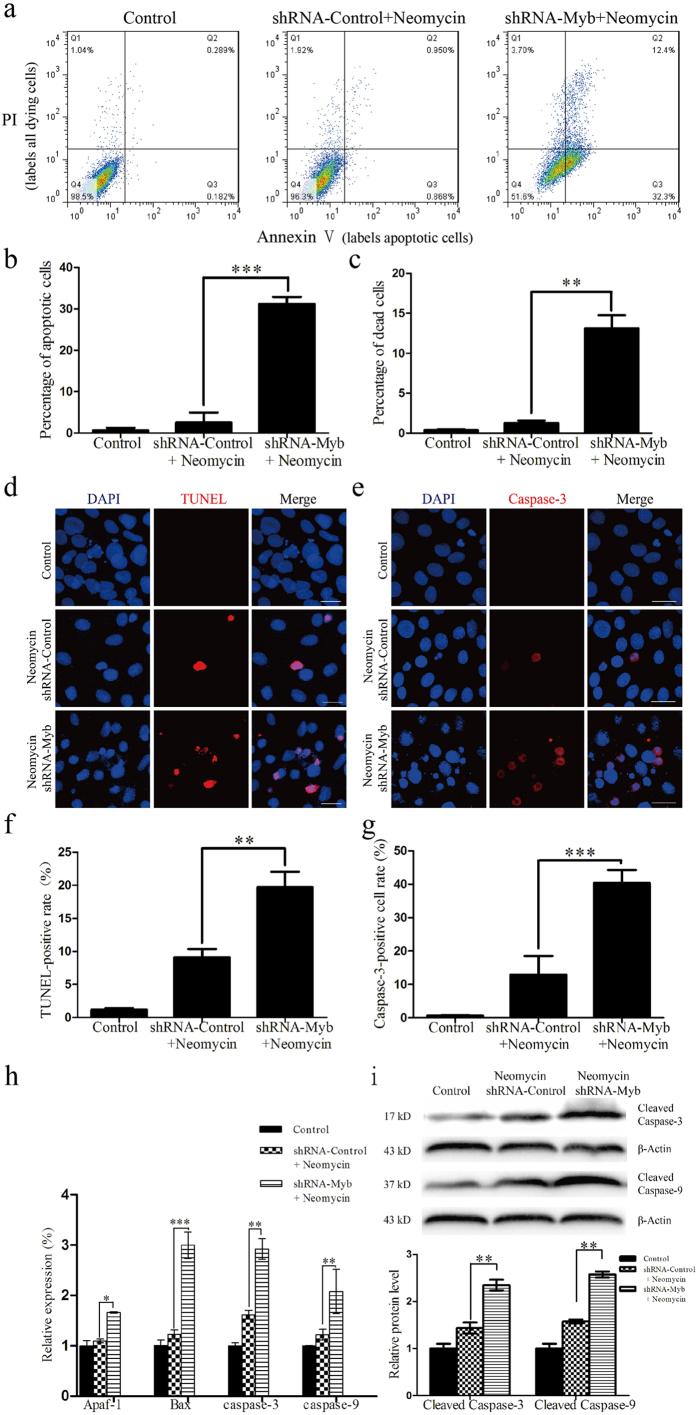
c-Myb knockdown increased HEI-OC1 cell apoptosis after neomycin treatment. All control groups were HEI-OC1 cells without any treatment. (**a–c**) Flow cytometry analysis in the control, shRNA-Control, and shRNA-Myb groups after neomycin exposure. Annexin V labels the apoptotic cells and propidium iodide (PI) labels all dying HEI-OC1 cells. (**d**) Representative images of TUNEL staining in the control, shRNA-Control, and shRNA-Myb groups after neomycin exposure. (**e**) Representative images of active caspase-3 staining in the control, shRNA-Control, and shRNA-Myb groups after neomycin exposure. (**f**) Quantification of TUNEL-positive cells in the control, shRNA-Control, and shRNA-Myb groups after neomycin exposure. (**g**) Quantification of active caspase-3–positive cells in the control, shRNA-Control, and shRNA-Myb groups after neomycin exposure. (**h**) qRT-PCR was used to measure the mRNA levels of Apaf-1, Bax, caspase-3, and caspase-9 in the control, shRNA-Control, and shRNA-Myb groups after neomycin exposure. (**i**) The western blot was used to measure the protein levels of cleaved caspase-3 and cleaved caspase-9 in the control, shRNA-Control, and shRNA-Myb groups after neomycin exposure. *p < 0.05, **p < 0.01, ***p < 0.001, n = 3. Scale bars = 30 μm.

At 48 h after transfection, the HEI-OC1 cells were treated with 2 mM neomycin for 24 h. We used flow cytometry analysis with Annexin V to label the apoptotic cells and propidium iodide (PI) to label the dead HEI-OC1 cells. The percentage of apoptotic cells was significantly increased in the shRNA-Myb transfected group (31.23 ± 1.68%) compared with the shRNA-Control group (2.51 ± 2.44%) after neomycin exposure (***p < 0.001, n = 3) (Fig. 6a,b). The percentage of dead cells was also significantly increased in the shRNA-Myb group (13.13 ± 1.63%) compared to the shRNA-Control group (1.28 ± 0.29%) after neomycin insult (**p < 0.01, n = 3) (Fig. 6a,c). Previous studies have reported that cleaved caspase-3 and TUNEL can be used as markers of apoptosis in aminoglycoside-induced HC death^30^,^32^,^33^,^34^,^35^,^36^. To confirm our findings, we performed active caspase-3 and TUNEL staining to detect the apoptotic HEI-OC1 cells. After neomycin exposure, the shRNA-Myb–transfected groups had a higher percentage of TUNEL-positive cells (19.69 ± 2.36%) compared to the shRNA-Control–transfected groups (9.09 ± 1.28%, **p < 0.01, n = 3) (Fig. 6d,f). The proportion of caspase-3-positive cells was also significantly increased in the shRNA-Myb groups (40.41 ± 3.89%) compared to the shRNA-Control groups (12.95 ± 5.55%, ***p < 0.001, n = 3) (Fig. 6e,g). These results showed that c-Myb knockdown significantly increased apoptosis in HEI-OC1 cells after neomycin exposure. We further investigated the possible mechanism behind this observation by measuring the activities of genes relevant to apoptosis. We found that the mRNA levels of Apaf-1, Bax, caspase-3, and caspase-9 in shRNA-Myb–transfected cells were 1.66 ± 0.02 fold, 3.00 ± 0.26 fold, 2.92 ± 0.20 fold, and 2.08 ± 0.44 fold greater, respectively, compared to the shRNA-Control group (*p < 0.05, **p < 0.01, ***p < 0.001, n = 3) (Fig. 6h).

Western blot results confirmed that the protein levels of cleaved caspase-3 and cleaved caspase-9 were increased in the shRNA-Myb–transfected group by 2.35 ± 0.11 fold and 2.57 ± 0.06 fold, respectively, compared to the shRNA-Control group (**p < 0.01, n = 3) (Fig. 6i).

### Knockdown of c-Myb expression increases HEI-OC1 cell apoptosis via down-regulation of Bcl-2

**Figure 7:**
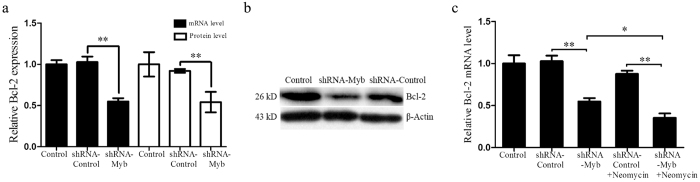
c-Myb knockdown decreased Bcl-2 expression in HEI-OC1 cells. All control groups were HEI-OC1 cells without any treatment. (**a**) The mRNA and protein levels of Bcl-2 in the control, shRNA-Control and shRNA-Myb groups. (**b**) Western blot results showed the protein levels of Bcl-2 in the control, shRNA-Control, and shRNA-Myb groups at 48 h after transfection without neomycin injury. (**c**) The mRNA levels of Bcl-2 in the control, shRNA-Control, shRNA-Myb, shRNA-Control-neomycin, and shRNA-Myb-neomycin groups. *p < 0.05, **p < 0.01, n = 3.

Bcl-2 suppresses apoptosis via its interaction with other pro-apoptotic members of the Bcl-2 family, and it is reported that c-Myb can regulate Bcl-2 expression^22^. We therefore examined the mRNA and protein levels of Bcl-2 in transfected HEI-OC1 cells without neomycin treatment. In the shRNA-Myb–transfected cells, the relative mRNA level of Bcl-2 decreased to 0.55 ± 0.04 that of the control and the relative protein level of Bcl-2 decreased to 0.54 ± 0.12 that of the control, suggesting that as the expression level of c-Myb decreased, there was a corresponding down-regulation of Bcl-2 expression in HEI-OC1 cells (**p < 0.01, n = 3) (Fig. 7a,b). In a separate experiment, at 48 h after transfection, we treated HEI-OC1 cells with 2 mM neomycin for 24 h. We found that after neomycin treatment, the relative mRNA level of Bcl-2 in the shRNA-Myb group was further reduced to 0.35 ± 0.05 that of the control (*p < 0.05, **p < 0.01, n = 3) (Fig. 7c). Taken together, these results demonstrated that c-Myb knockdown significantly down-regulated Bcl-2 expression, which could lead to the increased apoptosis seen in HEI-OC1 cells after neomycin injury.

### Knockdown of c-Myb expression increases intracellular ROS levels after neomycin injury

**Figure 8:**
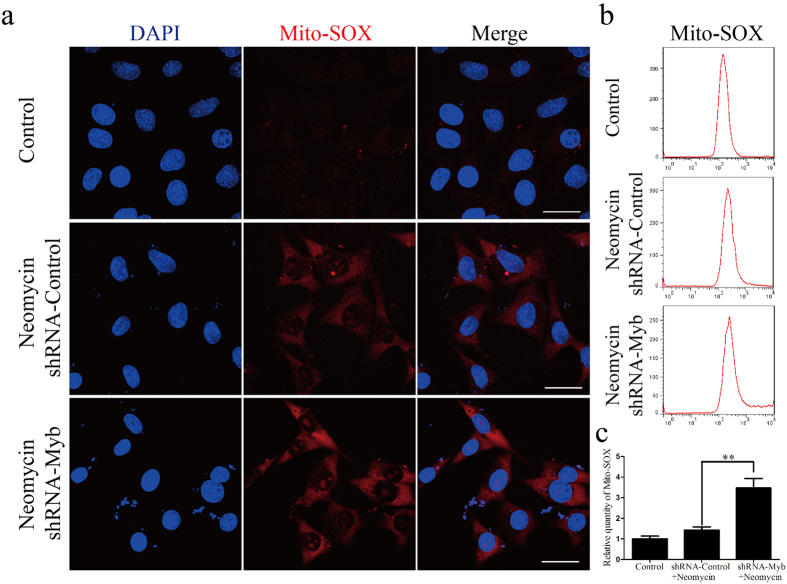
c-Myb knockdown increased the ROS level in HEI-OC1 cells after neomycin exposure. All control groups were HEI-OC1 cells without any treatment. (**a**) Representative images of Mito-SOX staining in the control, shRNA-Control, and shRNA-Myb groups after neomycin exposure. (**b**) Flow cytometry data confirmed the results in (**a**). (**c**) Quantification of the data in (**b**). *p < 0.05, **p < 0.01, ***p < 0.001, n = 3. Scale bar = 30 μm.

It is reported that ROS play important roles in aminoglycoside-induced HC damage^35^,^36^,^37^. We used Mito-SOX Red, which is a redox fluorophore that selectively detects mitochondrial superoxide, to evaluate ROS levels in transfected HEI-OC1 cells after neomycin injury. Immunohistochemistry and flow cytometry results showed that after neomycin treatment, the ROS level was significantly increased in HEI-OC1 cells transfected with shRNA-Myb (3.47 ± 0.46 fold compared to untreated controls) compared with the shRNA-Control group (1.42 ± 0.16-fold compared to untreated controls) (**p < 0.01, n = 3) (Fig. 8a–c). These results suggested that c-Myb knockdown significantly increases intracellular ROS levels after neomycin injury.

## Discussion

Previous studies have demonstrated the importance of c-Myb in cellular proliferation, differentiation, and apoptosis in the hematopoietic system, the mammary epithelium, the colonic epithelium, and certain other tissues. Betancur *et al*. used *in situ* hybridization to show that c-Myb is expressed within the chicken otic placode, which is a specialized region of ectoderm that gives rise to components of the inner ear. The combined action of the transcription factors c-Myb, Sox8, and Pea3 is required to initiate Sox10 gene expression, which serves as a key marker for otic development, thus showing that c-Myb has an important regulatory function in otic placode development^27^. However, the expression of c-Myb in the mammalian cochlea has not been studied previously.

We have shown for the first time that c-Myb exists in the mammalian cochlea and HEI-OC1 cells. In the postnatal organ of Corti, c-Myb is specifically expressed in the HCs and is up-regulated during later postnatal development. In the hematopoietic system, c-Myb is down-regulated after cell differentiation^16^,^17^,^18^, while in the aged hippocampus of gerbils c-Myb expression is significantly increased compared to the neonatal animals^19^. It has been reported that as a transcription factor, c-Myb might exert its effects by either activating or repressing genes in a cell type-specific manner^38^. Thus, we speculate that in the mammalian cochlea c-Myb is expressed in a HC-specific manner and might play a role in the maturation and differentiation of the postnatal cochlear sensory epithelium.

Aminoglycosides such as neomycin are widely used in clinics to treat bacterial infections, but ototoxic side effects greatly limit their clinical use^39^,^40^. In the current study, we found that in cochlear HCs c-Myb expression was decreased after neomycin exposure. In HEI-OC1 cells, c-Myb expression was reduced with the increasing time of neomycin exposure. These results indicate that c-Myb might play an otoprotective role in normal cochlear HCs.

To examine the effect of c-Myb on neomycin sensitivity, shRNA-Myb was generated and introduced into HEI-OC1 cells. We found that only HEI-OC1 cells transfected with shRNA-Myb exhibited a marked reduction in mRNA and protein levels of c-Myb, indicating that c-Myb is successfully down-regulated in HEI-OC1 cells. Next, we focused on exploring whether c-Myb knockdown had the potential to alter cell sensitivity to neomycin injury. We observed that c-Myb knockdown markedly reduced HEI-OC1 cell viability as demonstrated by the MTT assay, implying that endogenous expression of c-Myb has an important role in maintaining HEI-OC1 cell viability upon neomycin exposure.

Aminoglycosides have been widely reported to be ototoxic^39^ and to induce intrinsic apoptosis of HCs in birds and zebrafish through oxidative stress^8^,^9^. Our TUNEL assay and flow cytometry experiments showed significant cell apoptosis in the shRNA-Myb–transfected group after neomycin exposure. This suggested that after neomycin exposure, c-Myb knockdown decreases HEI-OC1 cell viability mainly via the induction of apoptosis. Caspases are the key proteins that modulate the apoptotic response. These activated caspases, especially, caspase-9 and caspase-3, can cleave many cellular substrates, eventually leading to apoptosis^41^. In the present study, we found that cleaved caspase-3 and cleaved caspase-9 were significantly increased in c-Myb knockdown cells after neomycin exposure, suggesting that the caspase-dependent pathway is activated in this process. Proteins of the Bcl-2 family are involved in the intrinsic apoptosis pathway and control the activation of the caspases^42^. Apoptotic protease-activating factor-1 (Apaf-1) can form a multi-protein complex with procaspase 9 and cytochrome c^43^,^44^, and is also involved in regulating apoptosis. Our work demonstrated that Bax and Apaf-1 were dramatically increased in c-Myb knockdown cells after neomycin exposure, while the expression of Bcl-2 was decreased, thus showing that c-Myb can regulate the expression of apoptosis-related genes in HEI-OC1 cells.

Mammalian sensory HCs have many mitochondria and high oxygen consumption, which makes them very sensitive to oxidative stress, especially when challenged by external stimulation such as noise or aminoglycosides^6^. Studies have confirmed that aminoglycoside-induced HC loss is commonly linked with mitochondrial dysfunction and the accumulation of ROS^45^,^46^, and c-Myb overexpression in cardiomyocytes results in lower levels of ROS^25^. In the present study, Mito-SOX staining demonstrated increased ROS levels in HEI-OC1 cells transfected with shRNA-Myb after neomycin treatment. This indicates that c-Myb knockdown in HEI-OC1 cells can increase cellular ROS levels after neomycin exposure.

The intrinsic apoptotic pathway is regulated by the pro-apoptotic factor Bax and the anti-apoptotic factor Bcl-2, and their combined action induces caspase-3–dependent apoptosis^47^,^48^. Previous studies have demonstrated that Bcl-2 is a target gene of c-Myb in many cell types^13^,^49^,^50^,^51^. However, whether regulation of Bcl-2 is a plausible mechanism for the protective function of c-Myb in HEI-OC1 cells is uncertain. In this work, we found that down-regulation of c-Myb in HEI-OC1 cells markedly decreased the expression of Bcl-2, suggesting that low activity of Bcl-2 might contribute to the enhanced sensitivity of c-Myb knockdown cells to neomycin exposure.

In conclusion, we provide the first report of the dynamic expression pattern of c-Myb in the mouse cochlea. We also demonstrate that c-Myb knockdown increases ROS levels and decreases Bcl-2 expression, which lead to the increased release of apoptotic factors from the mitochondria and enhance cell apoptosis of HEI-OC1 cells after neomycin injury. Our findings suggest that c-Myb might be a new therapeutic target for the prevention of aminoglycoside-induced HC death.

## Materials and Methods

### Animals and treatments

All animal experiments were approved by the Animal Care Committee of Shandong University, China, and followed the Guide for the Care and Use of Laboratory Animal for Research Purposes. C57BL/6 mice were purchased from the Animal Center of Shandong University (Jinan, China). To establish animal models, mice received a daily subcutaneous injection of neomycin (200 mg/kg) or sterile saline from P7 to P14.

### Cell line

The House Ear Institute-Organ of Corti 1 (HEI-OC1) cell line was derived from the cochlea of the Immortomouse and was characterized by Kalinec *et al*.^28^. Cells were maintained in high-glucose Dulbecco’s modified Eagle’s medium (DMEM; Gibco) supplemented with 10% fetal bovine serum (FBS; Gibco) at 33 °C in a humidified incubator with 5% CO_2._

### Cochlea dissection

C57BL/6 mice at P1, P7, P11, P14, and P30 were decapitated, and the temporal bones were collected and placed into cold sterile Hank’s Balanced Salt Solution (HBSS, Hyclone, USA). The stria vascularis, modulus, and tectorial membrane were removed with fine forceps under a microscope, and whole-mount cochleae were then placed onto 10 mm coverslips pre-coated with Cell-Tek (BD Biosciences, NJ, USA). These cochleae were used for determining c-Myb expression.

### Cryosectioning

Cochleae from C57BL/6 mice were removed and fixed with 4% paraformaldehyde in PBS at 4 °C overnight. After decalcification, tissues were cryoprotected by successive incubation in 10%, 20%, and 30% sucrose in 1 × PBS, embedded in O.C.T compound (Tissue-Tek, Sakura Finetek, USA), adjusted for the proper orientation, snap frozen on dry ice, and then stored at −80 °C overnight. Frozen sections were cut into 7 μm sections using a cryostat (Leica CM 1850, Nussloch, Germany).

### Immunostaining

Samples were permeabilized with 1% Triton X-100 in PBS (Sigma, USA). Nonspecific binding was blocked by incubation for 1 h in 0.1% Triton X-100, 5% donkey serum, 1% bovine serum albumin, and 0.02% sodium azide in PBS (PBT1). Tissues were then incubated overnight at 4 °C in PBT1 with the following primary antibodies: anti-c-Myb (1:1000 dilution, Millipore, USA), anti-cleaved caspase-3 (1:1000 dilution, Cell Signaling Technology Inc, USA), anti-myosin 7 A (1:1000 dilution, Proteus Biosciences, USA), and anti-Sox2 (1:500 dilution, Santa Cruz Biotechnology, USA). The next day, tissues were incubated with FITC-conjugated or TRITC-conjugated secondary antibody (1:1000 dilution, Invitrogen, USA) along with DAPI (1:800 dilution, Sigma-Aldrich, USA) in 0.1% Triton X-100 and 1% bovine serum albumin in PBS at room temperature for 1 h. The coverslips were mounted and observed under a laser scanning confocal microscope (Leica, Germany).

The TUNEL kit (Click-iT Plus TUNEL Assay for *In situ* Apoptosis Detection, Invitrogen, USA) was used to detect apoptotic cells according to the manufacturer’s instructions. Mito-SOX Red (Life Technologies, Waltham, USA) was used to detect ROS. HEI-OC1 cells pretreated were exposed to 2 mM neomycin for 24 h, then washed with PBS and incubated with Mito-SOX Red for 10 min at 37 °C. Cells were washed with preheated PBS and imaged by confocal microscope (Leica, Germany).

### RNA extraction and qRT-PCR

Total RNA was extracted from mouse cochleae and HEI-OC1 cells according to the manufacturer’s protocol using TRIzol Reagent (Invitrogen, USA). The total RNA (1 μg) was reverse-transcribed to cDNA using random hexamers and superscript reverse transcriptase. The expression of several genes was examined by qRT-PCR using the SYBR green Master Mix kit and an Eppendorf AG 22331 PCR machine (Hamburg, Germany). Primer sets are described in Table 1. The PCR conditions were a pre-denaturation step at 95 °C for 4 min, 40 cycles of denaturation at 95 °C for 30 s, annealing at 60 °C for 45 s, and extension at 72 °C for 1 min, and a final extension at 72 °C for 10 min. The level of gene expression in each sample was normalized to the respective expression level, and the specificity of each PCR reaction was confirmed by melting curve analysis.

### Protein extraction and western blot analysis

The cochleae and HEI-OC1 cells were harvested and lysed with RIPA buffer (Protein Biotechnology, China) containing a protease inhibitor cocktail (Sigma, USA) for 30 min at 4 °C. The lysates were centrifuged at 12,000 × *g* for 10 min at 4 °C, and protein concentrations were calculated using the BCA Protein Assay Kit (Protein Biotechnology, China). A total of 40 μg of each protein sample was denatured and separated by 10% SDS-PAGE. After electrophoresis, the separated proteins were transferred onto polyvinylidene difluoride membranes and were blocked with 5% non-fat dried milk in Tris-buffered saline and Tween 20 (TBST) for 1 h at room temperature. The membranes were then incubated with anti-c-Myb (1:1000 dilution, Millipore, USA), anti-Bcl-2 (1:500 dilution, Santa Cruz Biotechnology, USA), anti-β-actin (1:2000 dilution, ZSGB-BIO, China), anti-cleaved caspase-9 (1:500 dilution, Santa Cruz Biotechnology, USA), or anti-cleaved caspase-3 (1:1000 dilution, Cell Signaling Technology Inc, USA) antibodies overnight at 4 °C. Following three washes with TBST, the blots were incubated with secondary goat anti-mouse or goat anti-rabbit IgG antibody (1:2000 dilution, Abcam, UK) at room temperature for 1 h. Finally, the immunoblots were detected using an ECL kit (Santa Cruz Biotechnology, USA) and visualized after exposure to X-ray film. The relative optical density ratio was calculated with the Image J software by comparison to β-actin.

### shRNA transfection in HEI-OC1 cells

The c-Myb-specific shRNA (GenePharma, China) was designed to knock down the expression of c-Myb in HEI-OC1 cells. shRNA encoding a nonsense sequence was designed as the negative control, and nonsense shRNA conjugated with GFP was used to evaluate the transfection efficiency. Briefly, cells were seeded in 6- or 96-well plates at a density of 1 × 10^5^ cells/ml to ensure ~80% confluent cultures at 24 h after seeding. Lipofectamine 3000 (Thermo Fisher Scientific, USA) was added for transfection according to the manufacturer’s protocol with a DNA to Lipofectamine ratio of 1:3 w/v, and the P3000 enhancer reagent was used along with the Lipofectamine 3000 for all transfections. The Opti-MEM medium was replaced 8 h later with DMEM containing fetal bovine serum. After incubation at 33 °C and 5% CO_2_ for 48 h, cells were further treated with neomycin for 24 h. Samples were then prepared and analyzed. The shRNA sequences are listed in Table 2.

### Assessment of cell viability by MTT

For the measurement of cell viability, 3-(4,5-dimethyl-2-thiazoyl)-2,5-diphenyl-tetrazolium bromide (MTT) was used according to the manufacturer’s instructions (Roche, USA). HEI-OC1 cells with or without shRNA transfection were cultured in 96-well plates at a density of 1 × 10^5^ cells/ml and incubated with neomycin for 24 h. In the dose-dependence experiment, cells were pretreated with 1 mM, 1.5 mM, or 2.0 mM neomycin for 24 h. In the time-dependence experiment, cells were pretreated with 2 mM neomycin for 24 h or 48 h. MTT (5 mg/ml, 20 μl) was added to each well at 20 h or 44 h, respectively. After 4 h of incubation at 33 °C, the medium was removed and 100 μl DMSO was added into each well to dissolve the crystals. The optical density (OD) values were measured at 570 nm using an ELISA reader (Multiscan MK3). Relative cell viability was calculated according to the following formula: Relative cell viability (%) = (OD_experiment_ − OD_blank_)/(OD_control_ − OD_blank_) × 100%.

### Flow cytometry

Apoptosis was determined by flow cytometry using an Annexin V kit (BD). Briefly, HEI-OC1 cells with shRNA transfection were treated with 2 mM neomycin for 24 h. Cells without any treatment were used as the control group. The cells were trypsinized, collected by centrifugation at 3000 × *g* for 5 min, and then washed twice with PBS and re-suspended in 1 × binding buffer at a concentration of 1 × 10^6^ cells/ml. Annexin V-FITC and propidium iodide were added and gently mixed with the cells. After incubation for 15 min at room temperature in the dark, the cells were immediately analyzed by flow cytometry.

### Statistical analysis

For each condition, at least three individual experiments were conducted. Data are presented as the mean ± SD. A two-tailed, unpaired Student’s *t*-test was performed when comparing two groups, and a one-way ANOVA followed by a Dunnett’s multiple comparisons test was used when comparing more than two groups. A *p*-value < 0.05 was considered statistically significant.

## Additional Information

**How to cite this article**: Yu, X. *et al*. c-Myb knockdown increases the neomycin-induced damage to hair-cell-like HEI-OC1 cells in vitro. *Sci. Rep.* **7**, 41094; doi: 10.1038/srep41094 (2017).

**Publisher's note:** Springer Nature remains neutral with regard to jurisdictional claims in published maps and institutional affiliations.

## Supplementary Material

Supplementary Fig 1

## Figures and Tables

**Table 1:** PCR primer sequences used in the experiments.

Gene	Forward sequence	Reverse sequence
GAPDH	GTATGACTCCACTCACGG	GGTCTGGCTCCTGGAAGA
Caspase-3	GGAAGCGAATCAATGGACTCTGG	GCATCGACATCTGTACCAGACC
Caspase-9	GGACCGTGACAAACTTGAGC	TCTCCATCAAAGCCGTGACC
Bcl-2	ATCGCCCTGTGGATGACTGAGT	GCCAGGAGAAATCAAACAGAGGC
Bax	TCAGGATGCGTCCACCAAGAAG	TGTGTCCACGGCGGCAATCATC
c-Myb	AATTATCTGCCCAACCGG	AGACCAACGCTTCGGACC
Brn3.1	ACCCAAATTCTCCAGCCTACAC	GGCGAGATGTGCTCAAGTAAGT
Apaf-1	TGTGTGAAGGTGGAGTCAAGG	CCTCTGGGGTTTCTGCTGAA

**Table 2:** shRNA sequences used in the experiment.

shRNA	Sense	Antisense
shRNA-Control	CACCGTTCTCCGAACGTGTCACGTTTCAAGAGAACGTGACACGTTCGGAGAATTTTTTG	GATCCAAAAAATTCTCCGAACGTGTCACGTTCTCTTGAAACGTGACACGTTCGGAGAAC
Nonsense shRNA conjugated with GFP	CACCGCTCACTCAAGATTGTCAGCAATTCAAGAGATTGCTGACAATCTTGAGTGAGTTTTTTG	GATCCAAAAAACTCAAGATTGTCAGCAATCTCTTGAATTGCTGACAATCTTGAGTGAGC
shRNA-Myb	CACCGGAAATACGTGAACGCGTTCTTTCAAGAGAAGAACGCGTTCACGTATTTCCTTTTTTG	GATCCAAAAAAGGAAATACGTGAACGCGTTCTTCTCTTGAAAGAACGCGTTCACGTATTTCC
