# Supplementary material for: c-Myb knockdown increases the neomycin-induced damage to hair-cell-like HEI-OC1 cells in vitro
Source: Sci Rep. 2017 Jan 23;7:41094. doi: 10.1038/srep41094 (PMC5253735; doi:10.1038/srep41094)
Supplement: Supplementary Fig 1 [file srep41094-s1.pdf]

c-Myb knockdown increases the neomycin-induced damage to hair-cell-like HEI-OC1 cells in vitro

Xiaoyu Yu[a,b], Wenwen Liu[a,b], Zhaomin Fan[a], Fuping Qian[c,d], Daogong Zhang[a], Yuechen Han[a], Lei Xu[a], Gaoying Sun[a,b], Jieyu Qi[c,d], Shasha Zhang[c,d], Mingliang Tang[c,d], Jianfeng Li[a,b*], Renjie Chai[c,d*], Haibo Wang[a,b*]

[a] Otolaryngology-Head and Neck Surgery, Shandong Provincial Hospital Affiliated to Shandong University, Jinan, China

[b] Shandong Provincial Key Laboratory of Otology, Jinan, China

[c] Key Laboratory of Developmental Genes and Human Disease, Ministry of Education, Institute of Life Sciences, Southeast University, Nanjing 210096, China

[d] Co-innovation Center of Neuroregeneration, Nantong University, Nantong 226001, China

***Corresponding authors:**

Haibo Wang, M.D., Otolaryngology-Head and Neck Surgery, Shandong Provincial Hospital Affiliated to Shandong University, Jinan, P.R. China. Tel: +86-531-68777583. E-mail: whboto11@163.com.

And

Renjie Chai, Ph.D., Co-innovation Center of Neuroregeneration, Key Laboratory for Developmental Genes and Human Disease, Ministry of Education, Institute of Life Sciences, State Key Laboratory of Bioelectronics, Southeast University, Nanjing 210096, China, Tel/Fax: 86-25-83790971, E-mail: renjiec@seu.edu.cn

And

Jianfeng Li, Ph.D., Otolaryngology-Head and Neck Surgery, Shandong Provincial Hospital Affiliated to Shandong University, Jinan, China. Tel: +86-531-68777583. E-mail: lijianfeng@hotmail.com.

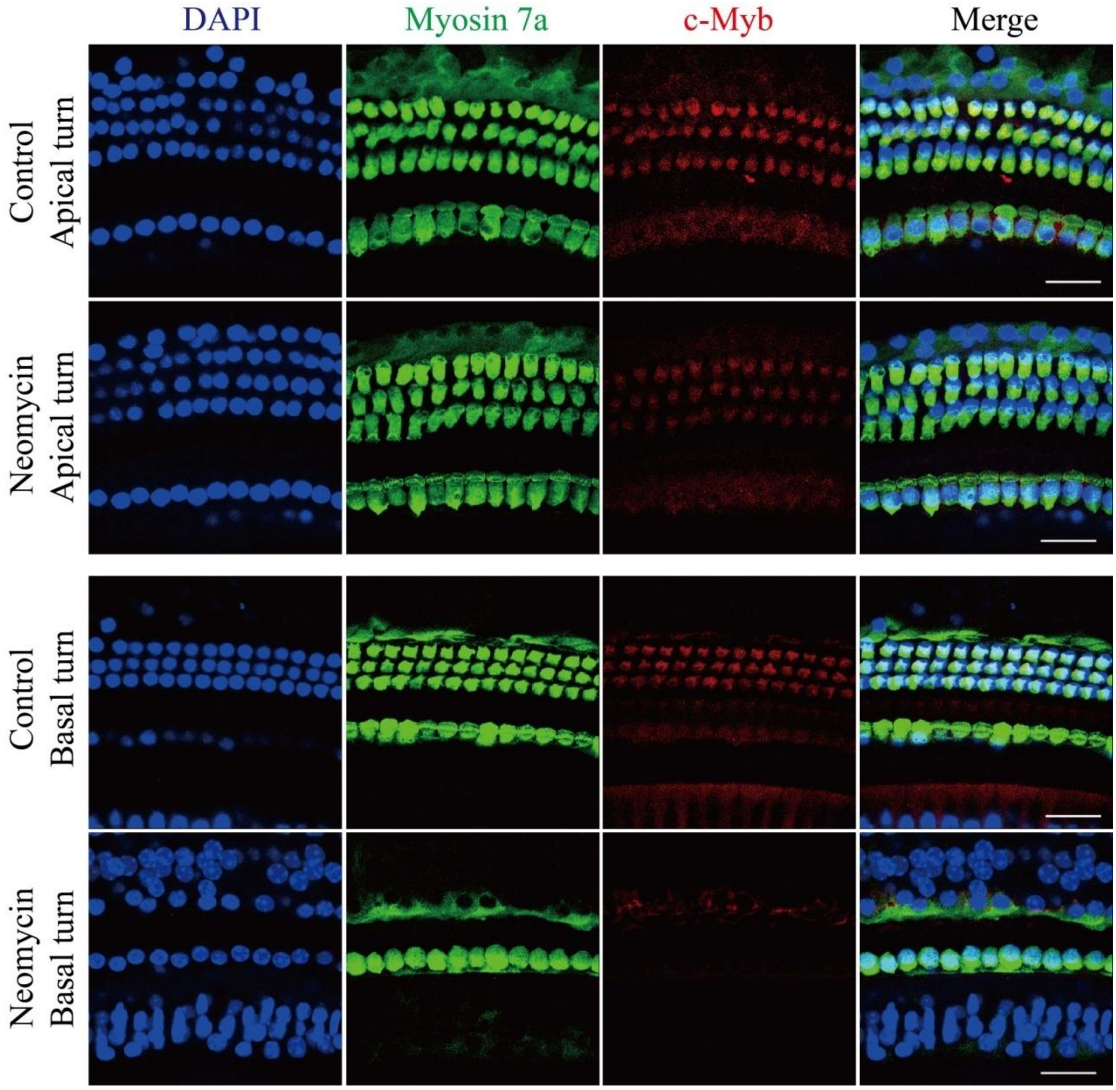

Supplemental figure 1. c-Myb expression was decreased in HCs of the apical and basal turns after neomycin exposure. Mice were given daily subcutaneous injections of neomycin (200 mg/kg) from P7 to P14. At P17, cochlear sensory epithelium samples were dissected out. Mice in the control group were given sterile saline. Scale bars = 30 μm.
